# Ion-shaping of embedded gold hollow nanoshells into vertically aligned prolate morphologies

**DOI:** 10.1038/srep21116

**Published:** 2016-02-17

**Authors:** Pierre-Eugéne Coulon, Julia Amici, Marie-Claude Clochard, Vladimir Khomenkov, Christian Dufour, Isabelle Monnet, Clara Grygiel, Sandrine Perruchas, Christian Ulysse, Ludovic Largeau, Giancarlo Rizza

**Affiliations:** 1 Laboratoire des Solides Irradiés, CNRS, CEA-DSM-IRAMIS, Ecole Polytechnique, Université Paris-Saclay, 91128 Palaiseau Cedex, France; 2 CIMAP-ENSICAEN-CEA-CNRS-University of Caen, Bd H. Becquerel, BP 5133, 14070, Caen Cedex 5, France; 3 Laboratoire de Physique de la Matière Condensée, CNRS, Ecole Polytechnique, Université Paris-Saclay, 91128 Palaiseau Cedex, France; 4 Laboratoire de Photonique et Nanostructures, CNRS, Marcoussis, France

## Abstract

Ion beam shaping is a novel technique with which one can shape nano-structures that are embedded in a matrix, while simultaneously imposing their orientation in space. In this work, we demonstrate that the ion-shaping technique can be implemented successfully to engineer the morphology of hollow metallic spherical particles embedded within a silica matrix. The outer diameter of these particles ranges between 20 and 60 nm and their shell thickness between 3 and 14 nm. Samples have been irradiated with 74 MeV Kr ions at room temperature and for increasing fluences up to 3.8 × 10^14^ cm^−2^. In parallel, the experimental results have been theoretically simulated by using a three-dimensional code based on the thermal-spike model. These calculations show that the particles undergo a partial melting during the ion impact, and that the amount of molten phase is maximal when the impact is off-center, hitting only one hemisphere of the hollow nano-particle. We suggest a deformation scenario which differs from the one that is generally proposed for solid nano-particles. Finally, these functional materials can be seen as building blocks for the fabrication of nanodevices with really three-dimensional architecture.

One of the priorities of the research activity in nano-science is the integration of nanostructures into functional matrices, circuits and chips. Over the past decade much effort has been put into pursuing this objective. This outburst of activity has mainly been triggered by technical progress, which has occurred both in the bottom-up approach (for instance, in the field of colloidal chemistry) and in the top-down approach. The latter has become feasible due to improvements in nano-structuration and deposition technologies. Although, this has opened the way to elegant methods for manufacturing planar nanostructures and nanodevices, it has remained a daunting task to engineer real three-dimensional architectures under the form of embedded nanostructures with tunable spatial orientation. One possible way to solve this longstanding problem could be using an ion beam as a tool to sculpt matter at the nanoscale. As a matter of fact, it has been well established that swift heavy-ion irradiation can be successfully put to use in order to elongate embedded nanoparticles (NPs) along the direction of the incoming ion beam. We will refer to this situation by stating with a metaphor colored by our personal interests that the “ion-shaping has been efficient”. By using this method, it has been possible to produce spatially oriented nanorods, nanowires and facetted metallic NPs with aspect ratios up to 30 inside several dielectric matrices^1^,^2^,^3^,^4^,^5^,^6^,^7^,^8^,^9^,^10^,^11^,^12^,^13^,^14^,^15^,^16^,^17^,^18^,^19^,^20^,^21^,^22^,^23^,^24^.

In the work we are reporting here, we have applied the ion-shaping technique to spherical hollow gold NPs (HNPs) embedded within a silica matrix. These HNPs consist of a cavity surrounded by a thin metallic shell of uniform thickness. In view of their unique optical properties, such HNPs have drawn the interest of many researchers. They are currently considered as ideal candidates for a wide range of biological and chemical applications, varying from catalysis to drug delivery, and from the treatment of cancer hyperthermia to surface-enhanced Raman spectroscopies^25^,^26^,^27^,^28^,^29^,^30^,^31^,^32^,^33^,^34^. However, the existing methods to synthesize them have remained limited to the fabrication of spherical HNPs. Here, we demonstrate the feasibility of using the ion-shaping technique to create a functional material that is composed of a dielectric thin film, which contains subsurface, vertically aligned prolate HNPs. Such a material can be considered as a building block for the fabrication of nanodevices with real three-dimensional architecture. The range of potential applications covers a whole spectrum from SERS substrates to thermoelectricity.

The manuscript is organized as follows: first, the ion-shaping of HNPs is explored systematically by not only varying their size but also their shell thickness. This thickness correlates of course with the dimension of their inner cavity. In parallel, the experimental results have been compared to the results of three-dimensional thermal-spike (3DTS) model simulations^21^. The latter have been used to determine how the molten fraction depends on the size and shell thickness of the HNPs, and on the offset parameter characterizing the location of the ion impact with respect to the center of the HNP. Finally, these results have been used to outline a mechanism that can explain the ion-shaping of HNPs. This mechanism is different from the one that is generally proposed for solid nanoparticles.

## Results and Discussion

**Figure 1:**
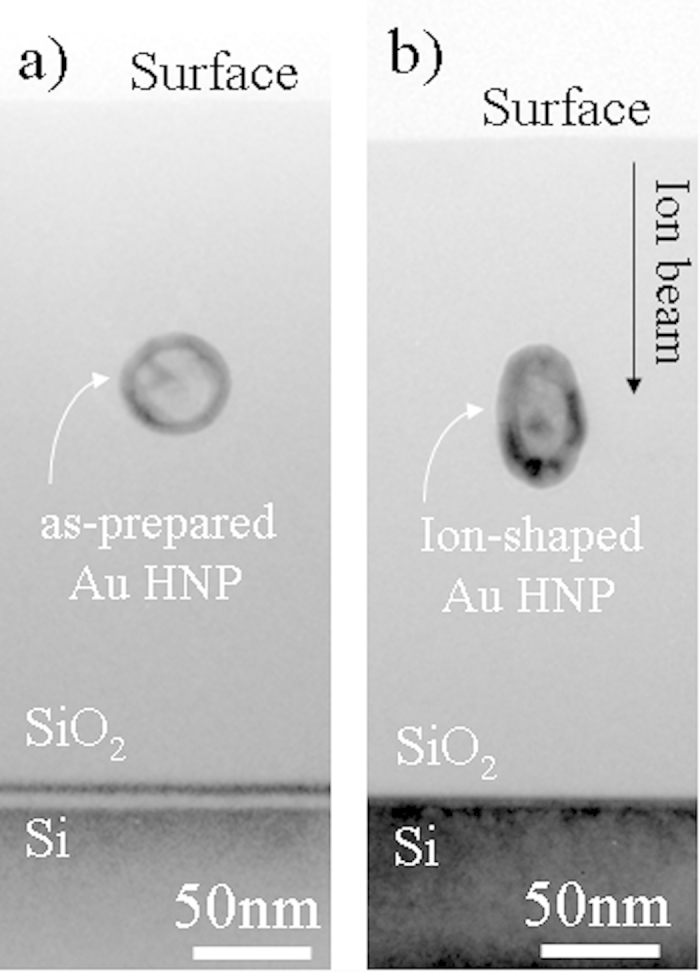
TEM micrographs showing (a) an as-prepared HNP embedded within the silica matrix, and (b) an ion shaped HNP after an irradiation at a fluence of 3.8 × 10^14^ at.cm^−2^ with 74 MeV Kr ions. The thickness of the irradiated silica matrix is reduced owing to the ion-hammering effect^36^.

An as-prepared sample is shown in Fig. 1(a). It shows a spherical HNP at 200 nm below the surface of the silica matrix. Figure 1(b) illustrates how the ion-irradiation can be used to shape the morphology of a nanostructure that is embedded in such a matrix. As can be seen the shape of the HNP has been transformed from spherical to prolate and the deformation takes place along the direction of the irradiating beam. The shape of the HNP is thus aligned with this direction which is normal to the sample surface. The sample was irradiated with 74 MeV Kr ions, and at the fluence of 3.8 × 10^14^ cm^−2^. The thicknesses of the silica matrices before and after the irradiation are different, which is due to the so-called *ion-hammering* effect^35^,^36^.

**Figure 2:**
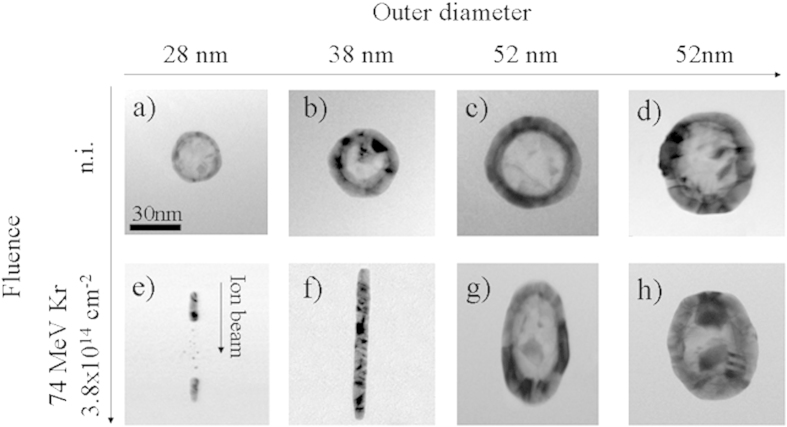
Matrix-like diagram showing the morphological evolution of Au HNPs for increasing initial outer diameter (x axis) and irradiation fluence (y axis). The ion-beam direction is indicated by the arrow.

However, the ion-shaping is not a straight process whereby all the HNPs are transformed in the same way. This is shown in Fig. 2 where a set of TEM micrographs are used to illustrate that the deformation pathway depends on both outer diameter and shell thickness of the embedded HNPs. For instance, three different behaviors can be identified for an irradiation at fluence of 3.8 × 10^14^ cm^−2^. First, Fig. 2(e) shows that a 28 nm HNP with a thin shell of 5 nm, Fig. 2(a), is not stable against irradiation and got fragmented into a combination of several small NPs (1–3 nm) and nanorods, which are all aligned along the beam direction. Second, Fig. 2(b) indicates that a 34 nm HNP with a thicker shell of about 10 nm transforms into a nanowire, Fig. 2(f). Here, the cavity has most likely been dissolved during the irradiation process. Third, Figs 1(h) and 2(g) describe the morphological deformation of two HNPs having nearly the same outer diameter (52 nm) but different shell thicknesses (8 nm and 12 nm), Figs 1(d) and 2(c). In both cases, cavity is conserved and deforms along with the surrounding metallic shell (Supplementary Information, S3). However, the efficiency of the ion-shaping becomes reduced when the shell thickness is increased.

**Figure 3:**
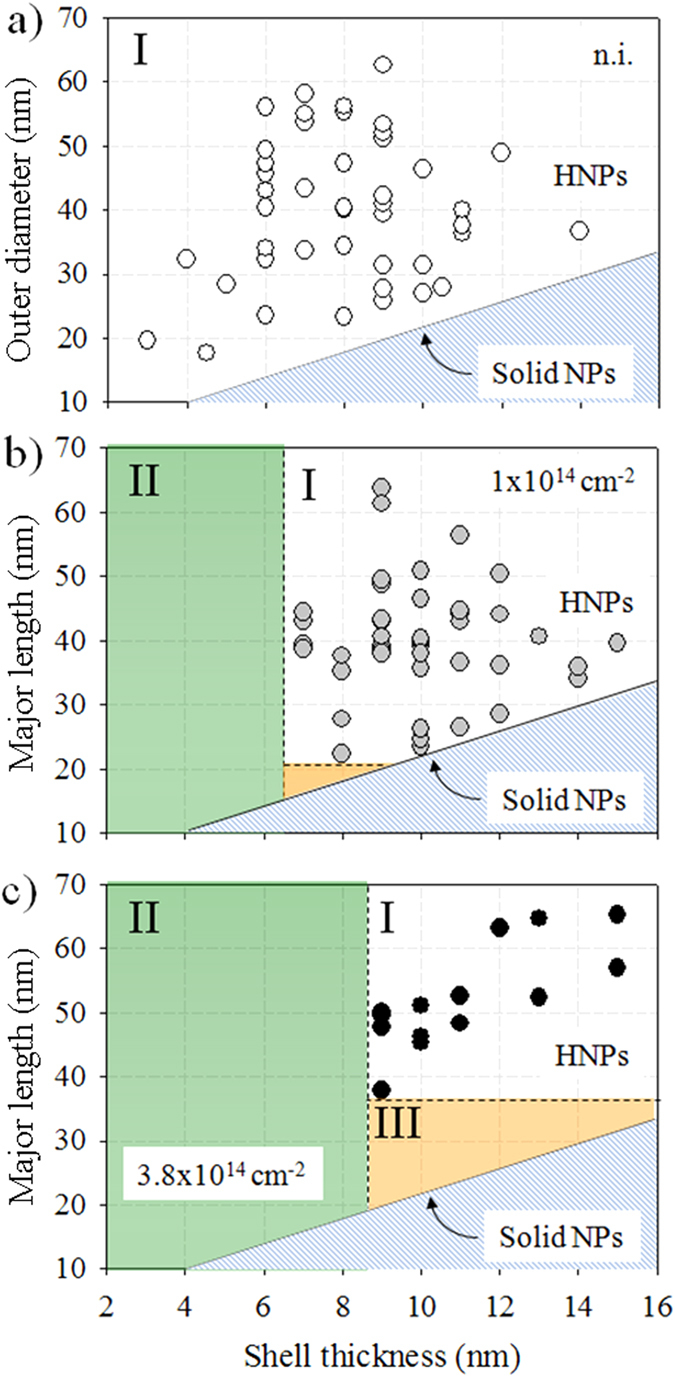
(**a**) The “phase diagram” of the ion-shaped HNPs in terms of their outer diameter and shell thickness can be subdivided into three regions. *Region I* encompasses the HNPs that are stable under irradiation and as such have been efficiently ion-shaped. *Region II* is characterized by HNPs that undergo explosion/fragmentation. *Region III* contains HNPs that are observed to elongate as solid NPs whereby the cavities are continuously dissolved under irradiation. The full black line corresponds to solid NPs. The evolution of the regions I, II, and III is shown for (**a**) the as-prepared HNPs, and for the HNPs irradiated at fluences of (**b**) 1 × 10^14^ cm^−2^, and (**c**) 3.8 × 10^14^ cm^−2^.

Even though Fig. 2 can be used to draw some general trends, visual observation of a limited number of HNP is not sufficient alone to give insights into the deformation process. Indeed, the phenomenology of the ion-shaping can only be described if the evolution of a statistical ensemble of HNPs is considered. A useful way to interpret the experimental results is to build a *phase-like diagram* where the outer diameter of the HNPs is represented as a function of shell thickness and irradiation fluence. This is shown in Fig. 3(a–c). The protocol we have used is described in more details in Supplementary Information, S4. Figure 3(a) represents the as-prepared sample. *Region I* in the diagram comprises a representative ensemble of chemically-synthesized HNPs. As these HNPs are nearly spherical in shape, they are characterized by their outer diameter, which ranges from 18 nm to 62 nm, and by their shell thickness, which ranges from 3 nm up to 14 nm. The lower boundary of *region I* (full black line) represents the condition for which the shell thickness is equal to its radius, i.e. a solid NP. Clearly, the region below this line has no physical meaning as the thickness of the shell can never be larger than the NP radius. Figure 3(b) shows how the system has evolved after a fluence of 1 × 10^14^ cm^−2^. It is worth noticing that elongated HNPs have a prolate shape, thus in Fig. 3(b–c) the outer diameter has been changed to their major length. Here, *region I* corresponds to the range of parameters for which HNPs are efficiently ion-shaped. It is readily apparent that only HNPs having a shell thicker than about 6 nm can be efficiently deformed, while a new region has emerged. This has been named *region II*. As a matter of fact, HNPs belonging to *region II* are unstable against irradiation and evolve toward fragmentation. For instance, their evolution in a similar to that shown in Fig. 2e). This being said, the frontier between *regions I and II* seems to depend only on the thickness of the metallic shell and not on the dimension of the HNPs. Also, a third region (colored orange in the figure) starts to develop (see below). Figure 3c) represents the evolution of the system after an irradiation fluence of 3.8 × 10^14^ cm^−2^. We observe that the extent of *region I* has been further reduced, in favor of the expansion of *regions II* and *III*. In particular, *region I* is now confined to the part of parameter space where outer diameters are larger than about 35 nm and shells thicker than about 8 nm. Besides, expansion of *region II* suggests a correlation between the stability of the HNPs, the thickness of the shell and the irradiation fluence. In particular, the larger the fluence the thicker the shell must be for the ion-shaping to be effective. In *region III* HNPs are observed to evolve into nanorods or nanowires, as shown in Fig. 2(f). Contrarily to *region II*, the extent of *region III* is function of both the size and the shell thickness of the HNPs.

**Figure 4:**
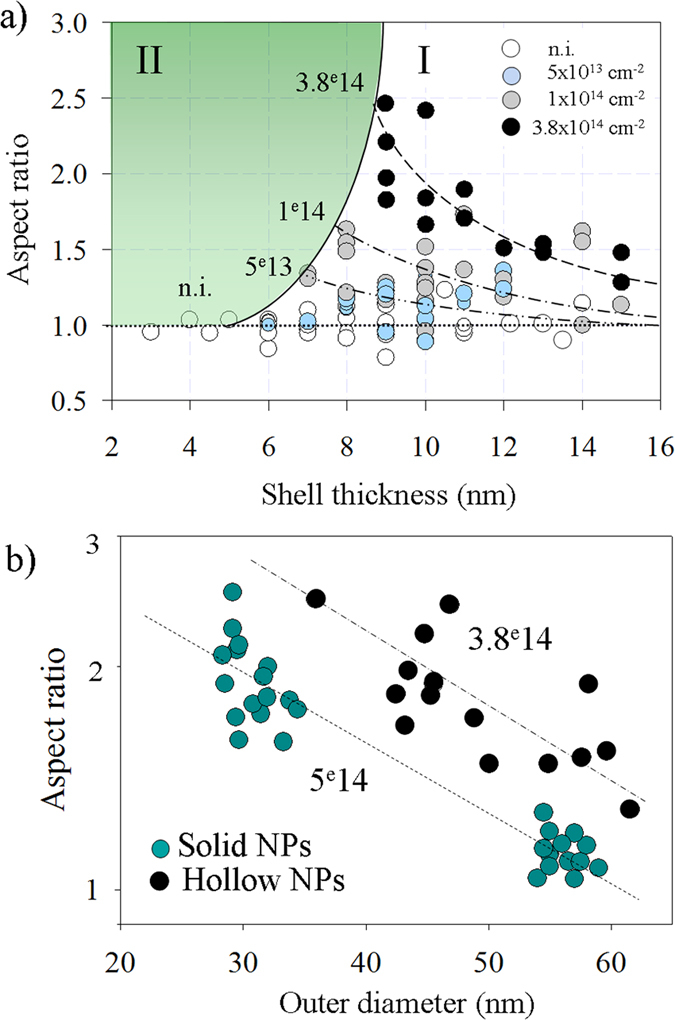
(**a**) The length-to-width aspect ratio is used to follow the deformation process with both the irradiation fluence and the thickness of the metallic shell. Dashed curves are to guide the eye and the continuous black curve separates region I, where HNPs are observed to be fragmented from region II, where HNPs are observed to be efficiently shaped. (**b**) Aspect ratio versus NP outer diameter for solid and hollow NPs.

Some insight into the kinetics of the ion-shaping of HNPs can be gained from an inspection of Fig. 4(a). Here, the length-to-width aspect ratio is used to characterize how the deformation depends on the irradiation fluence and on the thickness of the metallic shell. The as-prepared HNPs (open circles) are almost spherical, such that they are represented by points lying close to the line that corresponds to an aspect ratio of one. Besides, the deformation is observed to increase with the irradiation fluence, where two different regions (*I* and *II*) can be clearly identified: i) region *I* encompasses the HNPs that are stable against irradiation and have been efficiently ion-shaped. The aspect ratio increases with the irradiation fluence, while the ion-shaping efficiency rapidly decreases with the shell thickness. ii) Region *II* corresponds to HNPs that undergo explosion/fragmentation upon irradiation. The border that separates *region I* from *region II* defines the minimum shell thickness for which the HNPs are stable against irradiation. This parameter increases with the irradiation fluence from about 6 nm at 5 × 10^13^ cm^−2^ to about 8–9 nm at 3.8 × 10^14^ cm^−2^. In other words, the larger the irradiation fluence, the thicker the shell must be to allow for efficient shaping of the HNPs. It is worth noting that this value is close to a stability limit for the ion-shaped nanowires. It is the minimum width they can assume before a Rayleigh-like instability sets in, such that they break up and dissolve^9^. This point will be explained in more detail in the next section.

Finally, Fig. 4(b) indicates that HNPs are easier to deform than solid NPs and that despite being similar, the shaping mechanisms for these two types of particles are not strictly identical. Indeed, HNPs irradiated at a fluence of 3.8 × 10^14^ cm^−2^ exhibit a higher aspect ratio than solid NPs even when these have been irradiated at a higher fluence, i.e. 5 × 10^14^ cm^−2^.

### 3DTS simulations

**Figure 5:**
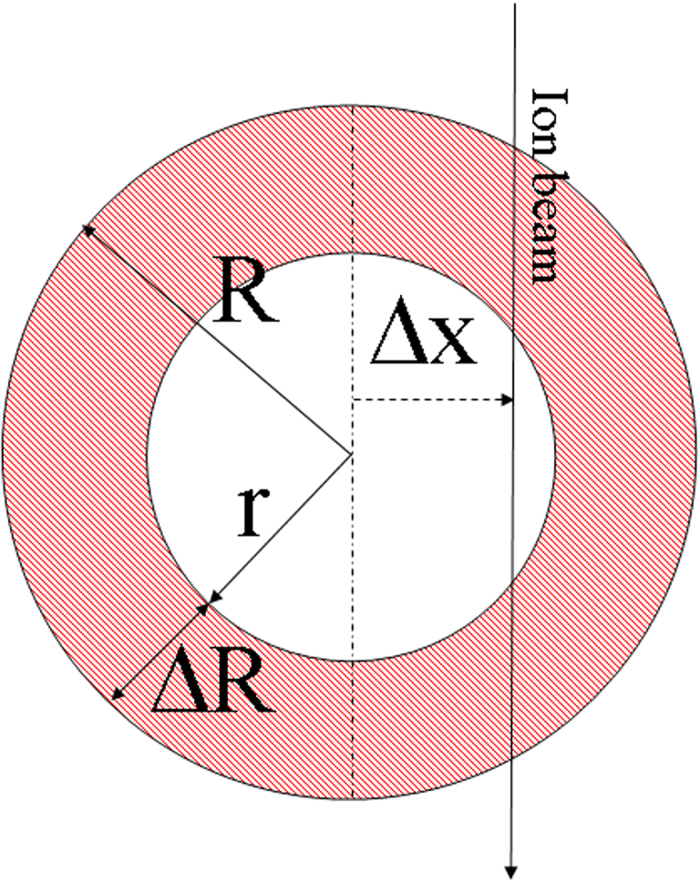
Conventions used to describe a HNP in the 3DTS code. *R*, *r* and Δ*R* = *R-r* are the outer radius, the cavity radius and the thickness of the silica shell, respectively. Δx is the position of the ion impact.

In a previous study we have proposed a possible ion-shaping mechanism for solid NPs^19^. It invokes the flow of a molten NP into a hot cylindrical region which develops in the wake of the projectile. In particular, the energy deposited into the metal’s electronic sub-system diffuses rapidly outwards toward the Au/silica interface, where it is efficiently transformed into heat. Successively, the molten phase propagates from the surface to the core of the NP. However, this mechanism has to be modified before it can be adopted in the present case, as by definition a HNP does not possess a core and the energy can only be deposited into the metallic shell. This is done by using the 3DTS code, whose details are described elsewhere^21^. For the sake of clarity, we have reported all the thermodynamic parameters used in the simulations in Table 1, and defined the meaning of the parameters used to describe the HNP-ion interaction in Fig. 5. Here, *R* represents the outer radius, *r* the radius of the cavity, Δ*r* = *R* − *r* the shell thickness and Δ*x* the offset distance of the impinging ion from the center of the HNP.

**Figure 6:**
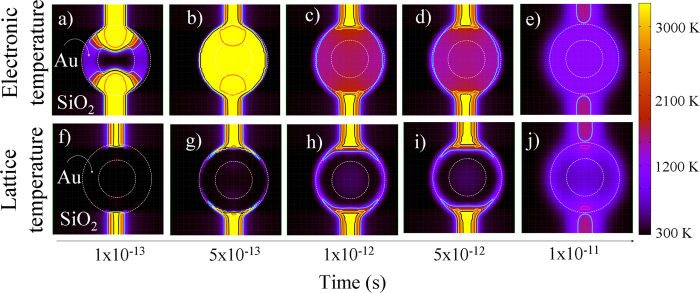
Timeline of the thermal evolution for a 42 nm Au HNP with a shell thickness of 7.5 nm embedded within a SiO_2_ matrix. Simulations have been run under the assumption that HNP is irradiated at normal incidence with a 74-MeV Kr ion. (**a–e**) Evolution of the electronic temperature. (**f–j**) Evolution of the lattice temperature.

Figure 6 is a timeline showing the thermal evolution of a HNP with R = 21 nm and Δ*R* = 7.5 nm after the passage of a 74-MeV Kr ion at Δ*x* = 0. It shows a series of calculated snapshots of cross-section views for increasing times in the interval between 10^−13^ and 10^−11^ s after the passage of the ion. During the ion-matter interaction, the energy of the impinging ion is mainly absorbed by the electrons of the target material. The corresponding temporal evolution of the electronic temperature is shown in the first row of the figure, i.e. Figure 6(a–e). Afterward, this energy is transferred to the atomic sub-lattice through electron-phonon interactions. This is represented in the second row, which shows the temporal evolution of the lattice temperature, Fig. 6(f–j). For times shorter than 5 × 10^−13^ s, a hot cylindrical region is formed within the silica matrix around the ion trajectory, i.e. the *latent track*. Meanwhile, the HNP remains solid, Fig. 6(f). For times larger than about 5 × 10^−13^ s, the HNP begins to melt, starting from its surface, Fig. 6(g). However, the molten phase remains localized close to the ion-impact, which in the study case chosen here corresponds to the poles of the HNP. For times longer than 5 × 10^−12^ s, the hot region within the shell of the HNP diffuses toward the equator. However, the temperature in this region does not reach the melting temperature, Fig. 6(h–i). Finally, the heat is dissipated and the HNP reaches the equilibrium for times larger than 1 × 10^−11^ s, Fig. 6(j).

**Figure 7:**
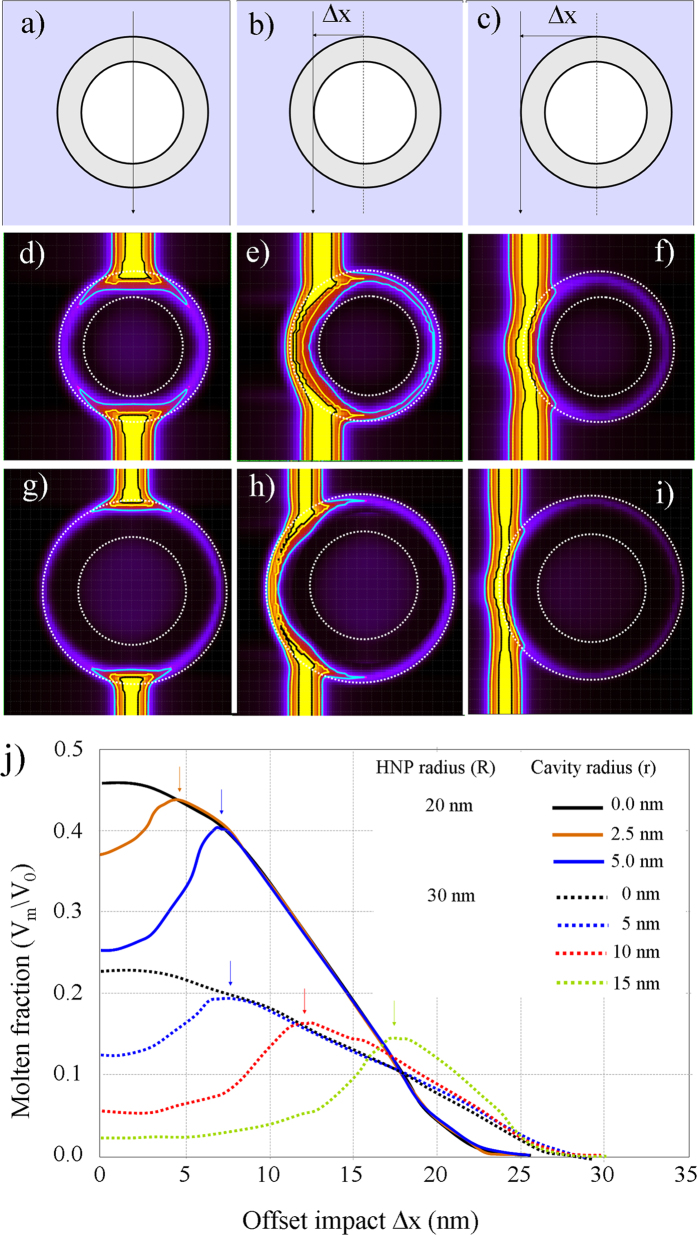
Sketches showing the ion-HNP interaction as a function of the distance Δ*x* of the ion trajectory from the NP center. (**a**) central impact, Δ*x* = 0, (**b**) impact at the edge of the cavity, Δ*x* = r, and (**c**) impact at the outer edge of the shell, Δ*x* = R. (**d–f**) 3DTS simulations for a HNP having an outer diameter of 21 nm and a shell thickness of 7.5 nm. (**g–i**) 3DTS simulations for a HNP having an outer diameter of 34 nm and a shell thickness of 12 nm. (**j**) Evolution of the molten fraction as a function of the impact offset distance Δx. This is shown for two different HNPs (R = 20 nm and 30 nm) and for several values of the cavity radius (or shell thickness). The maximum molten fraction is indicated by the arrows.

Next we analyze in detail the influence of the offset parameter, Δx, on the melting process of the HNP. The results for this ensemble of simulations are summarized in Fig. 7 and discussed hereafter. Sketches in Fig. 7(a–c) illustrate the initial conditions for three different values of Δx, which correspond to different types of ion impact: i) a central impact (Δ*x* = 0), ii) an impact at the edge of the cavity (Δ*x* = *r*), and iii) an impact at the edge of the HNP (Δ*x* = *R*). Figure 7(d–i) show the corresponding 3DTS simulations for two different HNPs. The first one has a outer radius and a shell thickness of 21 nm and 7.5 nm, respectively, Fig. 7(d–f). The second one has a radius of 34 nm and a shell of thickness 12 nm, Fig. 7(g–i). In both cases the part of the volume that melts becomes maximal when the ion passes in the vicinity of the edge of the cavity, i.e. for Δ*x* = *r*. However, the extend of this molten region decreases when the diameter of the HNP (or of the shell) increases.

Figure 7(j) shows how the molten-volume fraction is related to the offset parameter Δ*x*. Plots are shown for two different HNPs (R = 20 nm and 30 nm) and for several values of the cavity radius (or shell thickness). A first observation is that the molten fraction decreases when the shell becomes thinner as indicated by the arrows. This clearly demonstrates that the melting process depends on the amount of energy that is deposited within the NP. A second point is that the molten fraction decreases with the HNP size. For instance, fixing the cavity radius to *r* = 5 nm (solid and dashed blue lines), 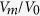 decreases from 0.4 to 0.2 when the outer radius of the HNP is increased from *R* = 20 nm to *R* = 30 nm. Finally, the molten fraction reaches its maximum value for impacts close to the edge of the cavity, Δ*x* = *r*. This is in contrast with what happens for solid NP (*r* = 0 nm), where the molten fraction reaches its maximum value for central impacts, Δ*x* = 0. This point can be understood by considering the ratio between the deposited energy *E*_*d*_ and the melting energy *E*_*m*_:


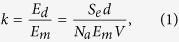


where, S_*e*_ is the electronic stopping power, N_*a*_ the atomic concentration, V = (4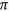/3)(R^3^ − r^3^) the volume of the HNP, and *d* the length of the ion path within the NP. The latter reads:


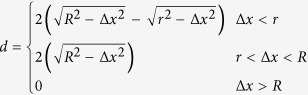


A simple calculation indicates that *k* is maximum when Δ*x* = *r*, i.e. for an ion passing at the edge of the cavity. However, in Fig. 7(k) this condition is not perfectly fulfilled. Indeed, 3DTS simulations indicate that the maximum is slightly shifted to the exterior of the metallic shell, i.e. to values of the impact offset parameter larger that the cavity radius, Δ*x *> *r*. This is due to the electronic-energy leakage, which, in turn, leads to the spreading of the heat outside the HNP^21^.

### Accounting for regions I, II and III

#### Region I

HNPs whose parameters fall into *region I* of Fig. 3 can be efficiently deformed (Fig. 7(g–h). Combining experimental data and 3DTS simulations we can suggest a possible mechanism for the ion-shaping of HNPs. We assume that the ion-shaping process requires a flow of metal into the liquid silica track and that this process is driven by the in-plane mechanical stress generated by the ion-hammering of the silica matrix.

**Figure 8:**
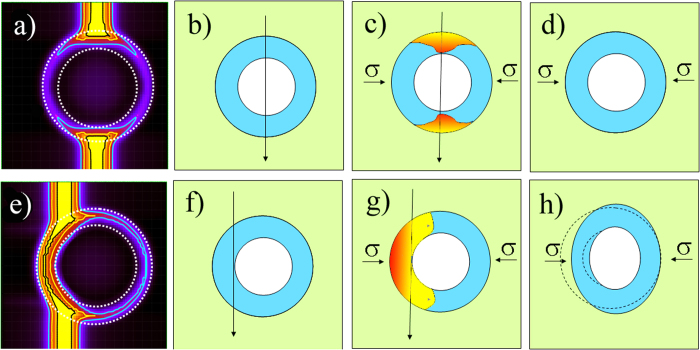
(**a**) 3DTS simulation for a central ion-impact. (**b–d**) Sketches showing why the ion-shaping is not effective for a central ion-impact. (**e**) 3DTS simulation for an offset ion impact (Δx = r). (**f–h**) Sketches showing the ion-shaping of the HNP for a non-central ion impact (Δx = r).

First, we show that for central impacts (Δ*x* = 0) the ion-shaping is not an effective process, Fig. 8(a–d). In this case, the molten phase is only localized around the two poles. The rest of the HNP remains in a solid phase while it is submitted to an in-plane stress due to the hammering effect. This stress can be quantified as^37^:


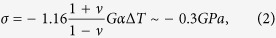


where 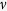 is the Poisson ratio, *G* the shear modulus and 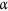 is the linear thermal-expansion coefficient. Klaumünzer has shown that for NPs in a solid phase the intensity of the in-plane stress, 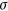, is not sufficient to drive the deformation of the NPs^38^. A good analogy would be to compare the HNP with an egg under pressure: it resists to the external pressure, and it is not deformed. For the ion-shaping to be effective, the impact must have an offset and take place at the edge of the cavity (Δ*x* = *r*), Fig. 8(e–h). This results in a maximal formation of molten phase within the HNP. However, the latter pervades only one hemisphere of the HNP. Here, the in-plane stress is exerted on a molten metallic shell, whose mechanical resistance to deformation is strongly reduced. Thus, the cavity is efficiently *deformed* whereby the metallic species diffuse toward the poles of the HNP. Thicker shells never melt completely. In this case the mechanical resistance to deformation is higher, which reduces the efficiency of the ion-shaping. This mechanism accounts for the fact that the deformation rate decreases when the shell becomes thicker.

#### Region II

HNPs whose parameters fall into *region II* of Fig. 3 are unstable against irradiation and undergo fragmentation, see e.g. Figure 2(e). 3DTS simulations indicate that shells thinner than about 8 nm are totally vaporized by the traversal of the impinging ion. In this case, the shell behaves as an ion-shaped NP reaching the minimal stability width. For gold this minimum occurs in the range 8–10 nm^20^. Here, Rayleigh-like instabilities develop in the wake of the projectile leading to the fragmentation/dissolution of the NP^9^.

#### Region III

**Figure 9:**
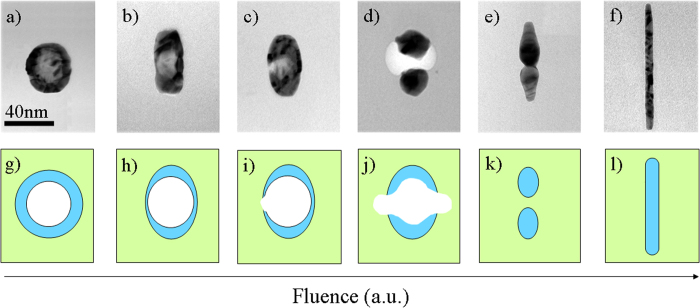
(**a**–**f**) TEM micrographs and (**g**–**l**) sketches depicting how a HNP can be transformed into a NW aligned along the ion beam.

HNPs whose parameters fall into *region III* of Fig. 3 are ion-shaped into nanowires aligned with the beam direction, while their cavities become dissolved, see e.g. Fig. 2f). This behavior can be understood with the aid of the TEM micrographs and the cartoons in Fig. 9(a–l). The as-prepared HNP has an homogeneous shell. However, during the irradiation the molten species diffuse toward the poles of the HNPs, reducing the shell thickness at the equator. When the latter is thinner than about 8 nm, the HNP becomes instable and undergoes fragmentation. This facilitates the leak of the cavity toward the surrounding silica matrix where it becomes instable. The latter mechanism is similar to the pore shrinkage observed in irradiated Vycor glasses^39^. This is in turn a manifestation of the relaxation of pressure in the hot part and shear stress in the fluid part of the ion track. We assume that the same mechanism is active here and that it leads to the compaction of the cavities from the moment on that they are extruded from the core of the HNP. After the complete dissolution of the cavity, the remaining fragments of the HNP can be efficiently shaped as solid NPs in the direction along the ion beam and eventually merge into a single nanowire. Finally, the fluence necessary to thin out the shell below the minimum width scales with both the shell thickness and the HNP dimension. This accounts for the fact that the extension of *region III* increases with the irradiation fluence, whereby larger HNPs necessitate a larger fluence for the cavity to be pushed out.

## Conclusion

In conclusion, in this work we have addressed the problem how to understand the ion-beam-shaping mechanism for the case of HNPs. We have shown that the ion-shaping does not only depend on the dimension of the HNP (as in the case of solid NPs). Also the thickness of the shell and the dimension of the cavity play a crucial role. Besides, 3DTS simulations have shown that the position of the ion-impact plays a predominant role in the ion-shaping of HNPs. The maximal amount of molten fraction one can obtain occurs indeed when the impinging ion passes close to the edge of the cavity. Under these conditions the molten phase remains spatially confined to one hemisphere of the HNP. The in-plane stress applied to the hot metallic shell cannot be compensated for by its surroundings as the center of the HNP is a cavity. This favors the squeezing of the shell and the deformation of the HNP. Using this approach, we are able to qualitatively account for the different experimental observations as a function of the HNP’s outer diameter, shell thickness and cavity dimension.

Finally, this work delineates the experimental limits for an efficient fabrication of functional materials formed by vertically aligned HNPs confined within a silica matrix. This represents the first step towards the fabrication of nano-composites with a really three-dimensional architecture.

## Methods

### Chemical synthesis

(Supplementary Information, S1) As already mentioned, HNPs consist of a cavity surrounded by a metallic shell. They are synthesized by galvanic replacement whereby cobalt NPs are used as sacrificial templates^27^,^28^. The protocol starts with the synthesis of cobalt colloids through the reduction of a cobalt salt in aqueous solution. In the next step, gold salt is added to the solution. The gold reduction (Au^3+^ → Au^0^) takes place as soon as the salt enters in contact with the surface of the cobalt NPs, triggering the growth of the gold shell while consuming the cobalt core. The overall synthesis is carried out under inert atmosphere following the protocol described hereafter. The solution is prepared in a round-bottomed flask, under inert (N_2_) atmosphere, by mixing 100 mL of deionized water with a sodium citrate (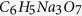) aqueous solution (0.1 Mol; 42.82 mg in 1 mL of deionized water), a fresh sodium borohydride (NaBH_4_) aqueous solution (1 Mol; 37.83 mg in 1 mL of deionized water) and a cobalt chloride (CoCl_2_) aqueous solution (0.5 Mol; 64.92 mg in 1 mL of deionized water). Afterward, the solution is kept under N_2_ atmosphere and magnetically stirred for 1 h. Successively, a certain number of 50 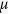
L doses of hydrogen tetrachloroaurate (HAuCl_4_) aqueous solution (0.1 Mol; 105 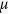
L in 5 mL of deionized water) were injected, whereby each time a waiting period of 1 min has been observed between successive injections. HNPs with different outer diameters, from 20 to 60 nm, and shell thickness, from 3 to 14 nm, have been synthesized by varying the reaction parameters. The as-synthesized HNPs are almost spherical with an aspect ratio between 1 and 1. 1, and with a homogeneous shell thickness. It may finally be noted that their structure is mainly polycrystalline, with grain sizes ranging from 5 to 15 nm.

### Chemical characterization

(Supplementary Information, S2) To check whether or not the sacrificial template, i.e. the Co NP, has been completely dissolved, the chemical composition of some HNPs has been determined by energy dispersive X-ray spectroscopy (EDS) on a probe-corrected JEOL 2200FS transmission electron microscope (TEM) operating at 200 kV. This has been done using both mapping and point-by-point modes. The data reveal the presence of significant amounts of gold (M 2.120 kV and L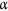 9.712 kV) from the HNPs, of oxygen and silicon (K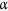 0.525 kV and K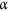 1.739 kV respectively) from the silica matrix and of copper (mainly K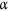 8.040 kV) due to the fluorescence from the microscope grid. The cobalt concentration (L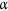 0.776 kV and K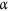 6.924 kV) however, is found to be below the detection limit of the EDX system (around 2%). This means that the cobalt core has been completely consumed by the gold reduction reaction before the formation of the gold shell was completed.

### Confinement of the HNPs

After their synthesis, the HNPs are confined within a silica matrix following the protocol reported hereafter, Fig. 1(a). This consists of four steps. First, a 200 nm thick silica layer is deposited by magnetron RF sputtering onto a silicon substrate. Second, the silica surface is functionalized using polyethylenimine (PEI) as a coupling agent. Then a drop of the solution containing the HNPs is deposited onto the sample surface, and the liquid solution is left to evaporate. Finally, the HNPs are embedded by depositing a second silica layer over them under the same conditions as used for the first one. As shown in Fig. 1(a), the embedding process does not modify the shapes of the HNPs which remain nearly spherical.

### Irradiation conditions

Irradiations have been performed at the GANIL facility (Caen, France) under normal incidence and at room temperature with 74 MeV Kr ions (0.86 MeV.u^−1^). Samples have been irradiated at the fluences of 5 × 10^13^, 1 × 10^14^ and 3.8 × 10^14^ cm^−2^. In order to avoid heating of the sample, the flux was kept constant at about 5 × 10^9^ cm^−2^.s^−1^. The electronic (S_*e*_) and the nuclear (S_*n*_) stopping powers were calculated with the SRIM2008 code^40^, both for the SiO_2_ matrix and for the Au NPs (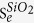 = 9.2 keV.nm^−1^, 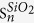 = 4 × 10^−2^ keV.nm^−1^, 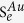 = 25.6 keV.nm^−1^, 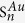 = 0.17 keV.nm^−1^). The evolution of the shape of the HNPS is investigated in cross-sectional geometry using a JEOL 2010F electron microscope operating at 200 kV at medium magnification (10-100 kx), see e.g. Fig. 1(a–b).

### Morphological characterization

(Supplementary Information, S4) For solid NPs - i.e. NPs with a solid core - calculating the relation between their initial size and their final ion-shaped morphology is a straightforward problem^19^. However, for HNPs this task is much more challenging, owing to the presence of the cavity. This problem is solved by comparing HNPs with the same amount of atoms within the metallic shell and the same mean shell thickness. In this case, the following relationships have been used for spherical and prolate HNPs, respectively:


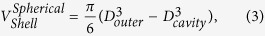



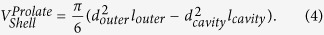


Here, 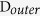 and 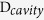 are the diameter of the spherical HNP and cavity, respectively. 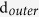 and 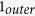 (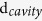 and 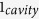) are the width and the length of the prolate HNP (cavity), respectively.

## Additional Information

**How to cite this article**: Coulon, P.-E. *et al*. Ion-shaping of embedded gold hollow nanoshells into vertically aligned prolate morphologies. *Sci. Rep*. **6**, 21116; doi: 10.1038/srep21116 (2016).

## Supplementary Material

Supplementary Information

## Figures and Tables

**Table 1:** Thermodynamic parameters used in the 3DTS simulations: 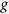 is the electron-phonon coupling, 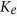 the electronic thermal conductivity, 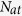 the atomic density, 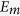 the melting energy, and S_*e*_ the electronic stopping power.

Element	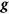 (W.cm^−3^.K^−1^)	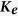 (W.cm^−1^.K^−1^)	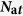 (10^22^cm^−3^)	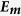 (eV.at^−1^)	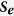 (keV.nm^−1^) 74 MeV Kr
Au	2.3 × 10^10 ^41	2.8^42^	5.90	0.433	26.3
SiO_2_	2.2 × 10^13 ^43	3 × 10^−5^T_*e*_	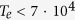 K^21^		9.2
		2.0,	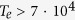 K		

## References

[b1] D′Orléans C. . Anisotropy of Co nanoparticles induced by swift heavy ions. Phys. Rev. B 67, 220101–220104(R) (2003).

[b2] Roorda S. . Aligned gold nanorods in silica made by ion irradiation of core-shell colloidal particles. Adv. Mater. 16, 235–237 (2004).

[b3] Penninkhof J. J., van Dillen T., Polman A., Graf C. & van Blaaderen A. Angle-dependent exinction of anisotropic silica/Au core/shell colloids made via ion irradiation. Adv. Mater. 17, 1484–1488 (2005).

[b4] Penninkhof J. J. . Anisotropic deformation of metallo-dielectric core-shell colloids under MeV ion irradiation. Nucl. Instr. Meth. Phys. Res. B 242, 52 (2006).

[b5] Mishra Y. K. . Synthesis of elongated Au nanoparticles in silica matrix by ion irradiation. Appl. Phys. Lett. 91, 063103 (2007).

[b6] Avasthi D. K., Mishra Y. K., Singh F. & Stoquert J. P. Ion tracks in silica for engineering the embedded nanoparticles. Nucl. Instr. Meth. B 268, 3027 (2010)

[b7] Amekura H. . Shape elongation of Zn nanoparticles in silica irradiated with swift heavy ions of different species and energies: scaling law and some insights on the elongation mechanism. Nanotechnology 25, 435301 (2014) 25288109 10.1088/0957-4484/25/43/435301

[b8] Dawi E. A., Rizza G., Mink M. P., Vredenberg A. M. & Habraken F. H. P. M. Ion beam shaping of Au nanoparticles in silica: particle size and concentration dependence. J. Appl. Phys. 105, 074305–074313 (2009).

[b9] Rizza G. . Rayleigh-like instability in the ion-shaping of Au-Ag alloy nanoparticles embedded within a silica matrix. Nanotechnology 22, 175305–175314 (2011). 21411926 10.1088/0957-4484/22/17/175305

[b10] Awazu K. . Elongation of gold nanoparticles in silica glass by irradiation with swift heavy ions. Phys. Rev. B 78, 054102–054109 (2008).

[b11] Oliver A. . Controlled anisotropic deformation of Ag nanoparticles by Si ion irradiation. Phys. Rev. B 74, 245425 (2006).

[b12] Reyes-Esqueda J. A. . Anisotropic linear and nonlinear optical properties from anisotropy-controlled metallic nanocomposites. Opt. Express 17, 12849 (2009). 19654691 10.1364/oe.17.012849

[b13] Rodriguez-Iglesias V. . Elongated Gold Nanoparticles Obtained by Ion Implantation in Silica: Characterization and T-Matrix Simulations. J. Phys. Chem. C 114, 746 (2010)

[b14] Giulian R. . Shape transformation of Pt nanoparticles induced by swift heavy-ion irradiation. Phys. Rev. B 78, 125413–125420 (2008). 10.1103/PhysRevLett.106.095505 21405636

[b15] Ridgway M. C. . Changes in metal nanoparticle shape and size induced by swift heavy-ion irradiation. Nucl. Instr. Meth. Phys. Res. B 267, 931–935 (2009).

[b16] Sprouster D. . Swift heavy-ion irradiation-induced shape and structural transformation in cobalt nanoparticles. J. Appl. Phys. 109, 113504–113510 (2011).

[b17] Rizza G., Dawi E. A., Vredenberg A. M. & Monnet I. Ion engineering of embedded nanostructures: From spherical to facetted nanoparticles. Appl. Phys. Lett. 95, 043105–043107 (2009).

[b18] Dawi E. A., Vredenberg A. M., Rizza G. & Toulemeonde M. Ion-induced elongation of gold nanoparticles in silica by irradiation with Ag and Cu swift heavy ions: track radius and energy loss threshold. Nanotechnology 22, 215607–215619 (2011). 21451236 10.1088/0957-4484/22/21/215607

[b19] Rizza G. . Rational description of the ion-beam shaping mechanism. Phys. Rev. B 86, 035450–035457 (2012).

[b20] Ridgway M. C. . Role of thermodynamics in the shape transformation of embedded metal nanoparticles induced by swift heavy-ion irradiation. Phys. Rev. Lett. 106, 095505–095508 (2011). 21405636 10.1103/PhysRevLett.106.095505

[b21] Dufour C., Khomenkov V., Rizza G. & Toulemonde M. Ion-matter interaction: the three-dimensional version of the thermal spike model. Application to nanoparticle irradiation with swift heavy ions. J. Phys. D: Appl. Phys. 45, 065302–065310 (2012).

[b22] Leino A. A., Djurabekova F. & Nordlund K. Radiation effects in nanoclusters embedded in solids. Eur. Phys. J. B 87, 242 (2014)

[b23] Ridgway M. C., Djurabekova F. & Nordlund K. Ion-solid interactions at the extremes of electronic energy loss: Examples for amorphous semiconductors and embedded nanostructures. Current Opinion in Solid State and Materials Science 19, 29 (2015)

[b24] Rizza G. From ion-hammering to ion-shaping: an historical overview. J. Phys.: Conf. Series 629, 012005 (2015).

[b25] Sun Y., Mayers B. T. & Xia Y. Template-engaged replacement reaction: a one-step approcah to the large-scale synthesys of metal nanostructures with hollow intersions. Nano Letters 2, 481–485 (2002).

[b26] Sun Y., Mayers B. & Xia Y. Metal nanostructures with hollow interiors. Adv. Mater. 15, 641–646 (2003).

[b27] Liang H. P., Wan L. J., Bai C. L. & Jian L. Gold hollow nanospheres: tunable surface plasmon resonance controlled by interior-cavity sizes. J. Phys. Chem. B 109, 7795–7800 (2005). 16851906 10.1021/jp045006f

[b28] Amici J. Preparation and characterization of metallic and metal oxide nanoparticles for biomedical applications. Politecnico Torino PhD Thesis 2013 doi: 10.6092/polito/porto/2511697.

[b29] Schwartzberg A. M., Olson T. Y., Talley C. E. & Zhang J. Z. Synthesis, characterization, and tunable properties of hollow gold nanoparticles. J. Phys. Chem. B 110, 19935–19944 (2006). 17020380 10.1021/jp062136a

[b30] Melancon M. P. . In vitro and *in vivo* targeting of hollow gold nanoshells directed at epidermal growth factor receptor for photothermal alation therapy. Mol. Cancer Ther. 7, 1730–1739 (2008). 18566244 10.1158/1535-7163.MCT-08-0016 PMC2727060

[b31] Lu W. . Effects of photoacustic imaging and photothermal ablation therapy mediated by targeted hollow gold nanospheres in an orthotopic mouse xenograft model of glioma. Cancer Res. 71, 6116–6121 (2011). 21856744 10.1158/0008-5472.CAN-10-4557 PMC3185126

[b32] Vongsavat V., Vittur B. M., Bryan W. W., Kim J.-H. & Lee T. R. Ultrasmall hollow gold-silver nanoshells whith extinctions strongly shifted to the near-infrared. ACS Appl. Mater. Interfaces 3, 3616–3624 (2011). 21761855 10.1021/am2008322

[b33] Zhang K. . Hollow gold nanospheres: growth morphology, composition and absorption characteristics. Micro Nanosyst. 3, 76–82 (2011).

[b34] Goodman A. M. . The surprising *in vivo* instabilty of near-IR-absorbing hollow Au-Ag nanoshells. ACS Nano 8, 3222–3231 (2014). 24547810 10.1021/nn405663h PMC4004326

[b35] Klaumünzer S. & Benyagoub A. Phenomenology of the plastic flow of amorphous solids induced by heavy-ion bombardment. Phy. Rev. B 43, 7502–7506 (1991). 10.1103/physrevb.43.7502 9996366

[b36] Klaumünzer S. . Ion-beam induced plastic deformation: A universal behavior of amorphous solids. Rad. Eff. Def. Sol. 108, 131–135 (1989).

[b37] Trinkaus H. Dynamics of viscoelastic flow in ion tracks: Origin of plastic deformation of amorphous materials. Nucl. Instr. Meth. Phys. Res. B 146, 204–216 (1998).

[b38] Klaumünzer S. Modification of nanostructures by high-energy ion beams. Nucl. Instr. Meth. Phys. Res. B 244, 1–7 (2006).

[b39] Klaumünzer S. Radiation compaction of porous Vycor glass. Nucl. Instr. Meth. Phys. Res. B 166/167, 459–764 (2000).

[b40] Ziegler J. F., Biersack J. P. & Littmark U. The Stopping Ranges and Ranges of Ions in Solids (Pergamon Press, New York, 1985).The simulation code is available at http://www.srim.org.

[b41] Wang Z., Dufour C., Paumier E. & Toulemonde M. The S_*e*_ sensitivity of metals under swift-heavy ion irradiation: a transient thermal process. J. Phys.: Condens. Mat. 6, 6733–6750 (1994).

[b42] CRC Handbook of Chemistry and Physics, 86th Edition (CRC Press, 2005).

[b43] Toulemonde M. . Synergy of nuclear and electronic energy losses in ion-irradiation processes: The case of vitreous silicon dioxide. Phys. Rev. B 83, 054106–054114 (2011).

