# Supplementary material for: Ion-shaping of embedded gold hollow nanoshells into vertically aligned prolate morphologies
Source: Sci Rep. 2016 Feb 17;6:21116. doi: 10.1038/srep21116 (PMC4756376; doi:10.1038/srep21116)
Supplement: Supplementary Information [file srep21116-s1.pdf]

Supplementary Information

Ion-shaping of embedded gold hollow nanoshells into vertically aligned prolate morphologies

Pierre-Eugène Coulon[1], Julia Amici[1], Marie-Claude Clochard[1], Vladimir Khomenkov[2,5], Christian Dufour[2], Isabelle Monnet[2], Clara Grygiel[2], Sandrine Perruchas[3], Christian Ulysse[4], Ludovic Largeau[4], and Giancarlo Rizza[1]

[1]Laboratoire des Solides Irradiés, CNRS, CEA-DSM-IRAMIS, Ecole Polytechnique, Université Paris-Saclay, 91128 Palaiseau Cedex, France
[2]CIMAP-ENSICAEN-CEA-CNRS-University of Caen, Bd H. Becquerel, BP 5133, 14070, Caen Cedex 5, France
[3]Laboratoire de Physique de la Matière Condensée, CNRS, Ecole Polytechnique, Université Paris-Saclay, 91128 Palaiseau Cedex, France
[4]Laboratoire de Photonique et Nanostructures, CNRS, Marcoussis, France
[5]Current address:Institute for Nuclear Research, 47 Nauky Ave., Kiev 03680, Ukraine

S1. Chemical synthesis

HNPs consist of a cavity surrounded by a metallic shell. They are synthesized by galvanic replacement whereby cobalt NPs are used as sacrificial templates. The protocol starts with the synthesis of cobalt colloids through the reduction of a cobalt salt in aqueous solution. In the next step, gold salt is added to the solution. The gold reduction ($Au^{3+} \rightarrow Au^{0}$) takes place as soon as the salt enters in contact with the surface of the cobalt NPs, triggering the growth of the gold shell while consuming the cobalt core. The overall synthesis is carried out under inert atmosphere following the protocol described hereafter. The solution is prepared in a round-bottomed flask, under inert (N_2) atmosphere, by mixing 100 mL of deionized water with a sodium citrate ($C_6H_5Na_3O_7$) aqueous solution (0.1 Mol; 42.82 mg in 1 mL of deionized water), a fresh sodium borohydride ($NaBH_4$) aqueous solution (1 Mol; 37.83 mg in 1 mL of deionized water) and a cobalt chloride ($CoCl_2$) aqueous solution (0.5 Mol; 64.92 mg in 1 mL of deionized water). Afterward, the solution is kept under N_2 atmosphere and magnetically stirred for 1h. Successively, a certain number of 50 mL doses of hydrogen tetrachloroaurate ($HAuCl_4$) aqueous solution (0.1 Mol; 105 mL in 5 mL of deionized water) were injected, whereby each time a waiting period of 1 min has been observed between successive injections. As summarized in Table SI, HNPs with different outer diameters and shell thickness can be synthesized by varying reaction parameters. TEM micrographs corresponding to the different classes of HNPs are shown in Fig. S1. It is readily apparent that they are almost spherical in shape, i.e. their aspect ratio ranges between 1 and 1.1, and they possess an homogeneous metallic shell. Finally, we note that their structure is mainly polycrystalline, with grain sizes ranging from 5 to 15 nm, see e.g. Fig. S3.

Class Size	$C_6H_5Na_3O_7$ (μl)	$NaBH_4$ (μl)	$CoCl_2$ (μl)	$HAuCl_4$ (μl)	Outer diameter (nm)	Inner diam. (nm)
1	400	100	100	250	32.9±5.2	21.6±3.0
2	330	250	100	450	34.6±8.7	22.1±4.1
3	330	250	100	700	53.3±8.0	40.1±2.3
4	900	100	100	450	55.6±9.7	37.6±5.1
5	190	180	100	450	59.4±6.2	48.4±5.3
6	190	180	100	700	63.9±7.6	49.2±6.1
7	900	180	100	700	63.9±5.6	46.8±3.6
8	400	100	100	700	68.5±8.7	44.1±6.7

Table SI: Protocols used to synthesize the different classes of HNPs

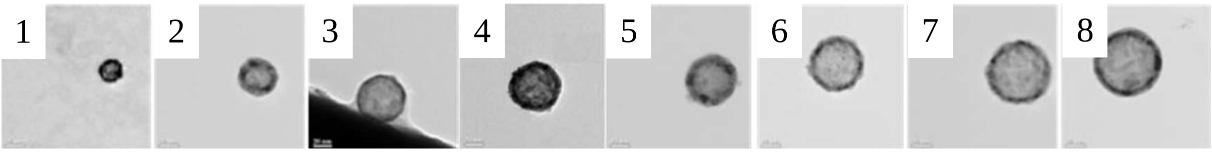

Fig. S1: Bright field TEM micrographs of the different classes of HNPs. Each class has been prepared following the receipt given in Table I.

S2. Chemical analysis (EDS)

Chemical analysis has been done, to check whether or not the sacrificial template, i.e. the Co NP, has been completely dissolved during the synthesis process. Chemical composition has been determined by energy dispersive X-ray spectroscopy (EDS) on a probe-corrected JEOL 2200FS transmission electron microscope (TEM) operating at 200 kV. This has been done using both mapping, Fig. S2b-c), and point by point modes, Fig. S2d-e). The data reveal the presence significant amounts of gold (M 2.120 kV and L_α 9.712 kV) from the HNPs, of oxygen and silicon (K_α 0.525 kV and Kα 1.739 kV respectively) from the silica matrix and of copper (mainly K_α 8.040 kV) due to fluorescence from the grid. On the contrary, the concentration of cobalt (L_α 0.776 kV and Kα 6.924 kV) is found to be below the detection limit of the EDX system (around 2%). This means that the cobalt core has been completely consumed by the gold reduction reaction before the formation of the gold shell was completed.

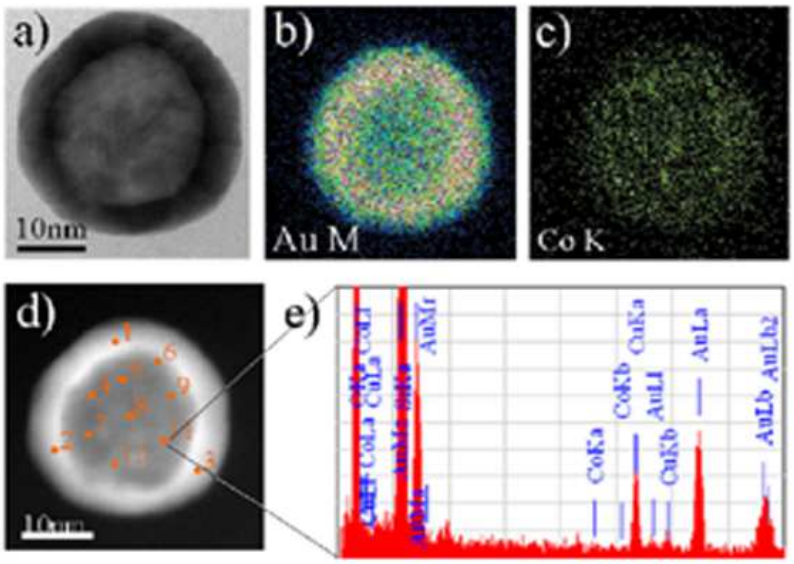

Fig. S2. a) Bright field TEM micrograph of a HNP. Chemical map for b) gold and c) cobalt. d) High Angle Annular Dark Field (HAADF) micrograph of a HNP. The numbered points indicate the positions where the EDS analysis has been performed. e) corresponding EDS spectrum for a local chemical analysis (point 11).

S3. Homogeneity and stability of the HNPs shell (HAADF analysis)

Homogeneity of the HNP shell and its stability against irradiation has been checked using Z-contrast technique. Here, an High Angle Annular Dark Field (HAADF) image is formed by collecting scattered electrons with an annular dark-field detector. In particular, the

intensity of an HAADF image in STEM is function of both the atomic number of the species in the sample (Z-contrast images) and the thickness of the specimen. In this work Z-contrast analysis was performed on a probe-corrected JEOL 2200FS microscope operating at 200 kV. Our system is composed of (heavy) gold HNPs (Z_{Au}=79) embedded within a (light) SiO_2 matrix (Z_O=8 and Z_{Si}=14), thus HNPs appear bright on a dark background as shown e.g. in Fig. S3b).

Stability of the shell against irradiation is confirmed by the fact that the contrast of the irradiated HNP, e.g. Fig. S3d), is similar to that observed in the as-prepared one, e.g. Fig. 3b). Indeed, a broken shell would result in a dark contrast similar to that of the background.

Sometimes, dark spots are sometimes observed in bright field images - a couple of examples are designated by the arrows in Figs. S3a) and S3c). The homogeneous contrast observed in HAADF images suggests that they are due to the diffraction of nanograins composing the metallic shell. Indeed, HAADF is only marginally affected by the crystallographic orientation of the crystallites.

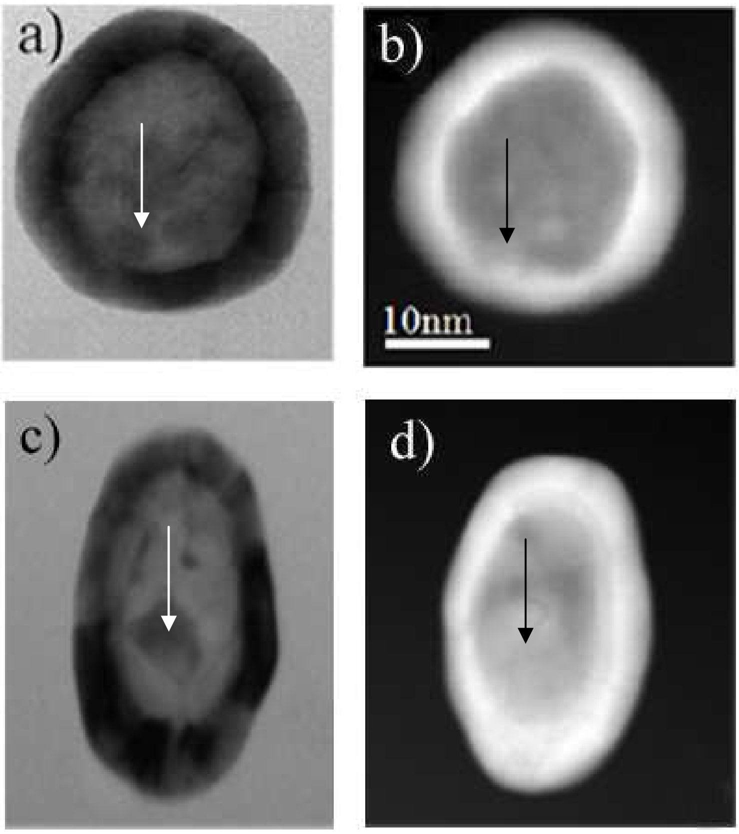

Fig. S3. a) Bright field and b) HAADF images of a non irradiated HNP. c) Bright field and d) HAADF images of an irradiated HNP. The arrows show nanograins in Bragg conditions indicating that the shell is polycrystalline.

S4. Construction of a "Phase-like diagram" for the ion-shaping of HNPs

Experimentally, an as-prepared spherical HNP is completely characterized by the dimensions of the outer (D_{Outer}) and inner (D_{Inner}) diameters, such that its volume and the thickness of the metallic shell can be written as :

$$V_{n.i.}^{HNP} = \frac{\pi}{6}\left(D_{Outer}^{3} - D_{Inner}^{3}\right)$$

$$\Delta R = D_{Outer} - D_{Inner}$$

A useful way to interpret the experimental results is to represent the ensemble of the as-prepared HNPs in a D_{Outer}-ΔR phase-like diagram as shown in Fig. S4a). For instance, the coordinates of the red and blue full circles represent two HNPs having the same size (52 nm) but different shell thicknesses (8 nm and 12 nm). Please note that experimental data shown in Figure S4 are a representative set of analyzed HNPs. The whole study has been done considering for each class size, as given in Table SI, and for each irradiation fluence at least one hundred HNPs.

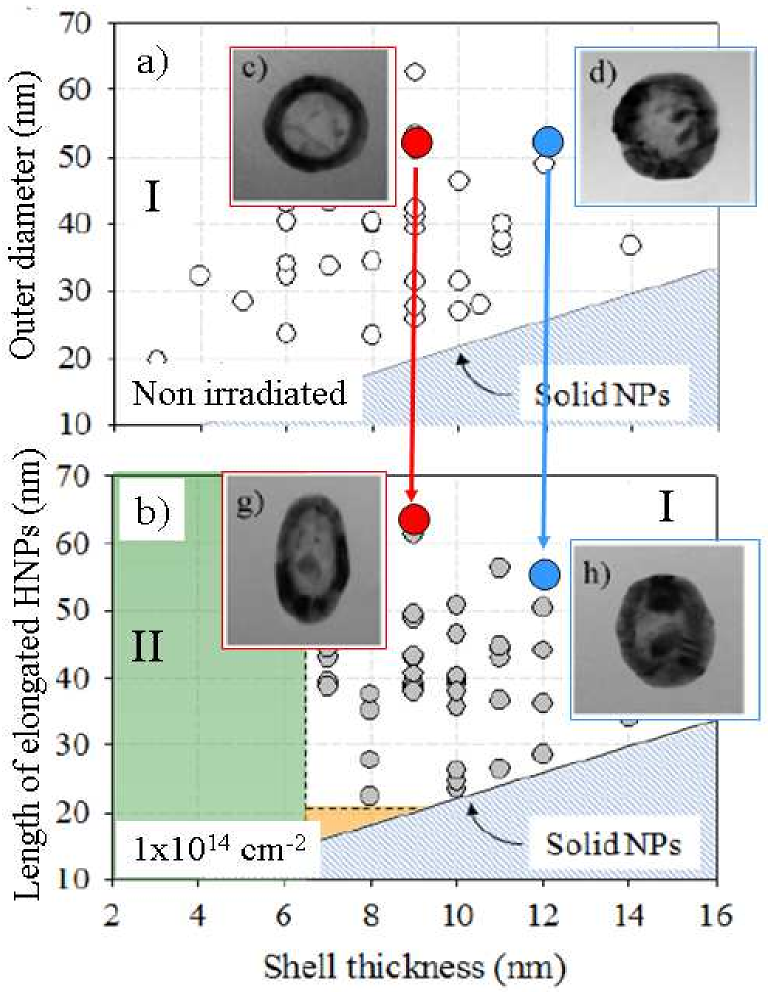

Fig. S4. Phase-like phase diagram and correlation between the non irradiated and the irradiated HNP

After irradiation, HNPs that have been efficiently deformed into prolate morphologies belong to region I of Fig. S4b). In this case, their volume and shell thickness write:

$$V_{Irr}^{HNP} \cong \frac{\pi}{6}\left(L_{Outer}W_{Outer}^{2} - L_{Inner}W_{Inner}^{2}\right)$$

$$\Delta d \cong W_{Outer} - W_{Inner}$$

$L_{Outer/Inner}$ are the outer and inner lengths and $W_{Outer/Inner}$ the outer and inner widths of the elongated HNPs. We note that in the Fig. S4b) the outer diameter has been changed to the length of the elongated HNP, $D_{Outer} \rightarrow L_{Outer}$.
The deformation pathway of the ion-shaped HNPs can be followed by assuming that both the volume and the shell thickness are conserved during the elongation process such that the following relationships hold:

$$V_{n.i.}^{HNP}(\Phi = 0) \cong V_{Irr}^{HNP}(\Phi)$$
$$\Delta R(\Phi = 0) \cong \Delta d(\Phi)$$

In this way, the red and blue arrows indicate the elongation pathways followed by the "red" and "blue" HNPs. Indeed, they point toward two ion-shaped HNPs having the same volume and the same shell thickness of the as-prepared ones (Figure S4b). Finally, this qualitative analysis indicates that for HNP of the same size, the efficiency of the (elongation) process is reduced when the thickness of the shell is increased.
